# Improving Therapeutic Ratios with the Oncotype DX® Ductal Carcinoma In Situ (DCIS) Score

**DOI:** 10.7759/cureus.1185

**Published:** 2017-04-21

**Authors:** Nafisha Lalani, Eileen Rakovitch

**Affiliations:** 1 Radiation Oncology, University of Toronto; 2 Sunnybrook Health Sciences Centre, University of Toronto

**Keywords:** dcis, breast cancer, genomic assay

## Abstract

Ductal carcinoma in situ (DCIS) is a non-invasive breast cancer comprising nearly 25% of breast cancer diagnoses in the mammographic era. Current guidelines recommend breast-conserving surgery followed by adjuvant radiotherapy; however, controversy exists regarding the appropriateness of these recommendations. Some women with DCIS will never recur, which raises the concern of over-treatment. Conversely, a small number of women will develop invasive recurrences, raising concerns of under-treatment. Currently, several clinical and pathologic factors have been identified as prognostic markers for recurrence; however, these variables alone have been unable to identify low-risk and high-risk subgroups. The Oncotype DX® DCIS score is a multigene assay which allows for the addition of molecular information to traditional clinical and pathologic factors to help guide treatment decisions. Here, we present two case examples illustrating the use of the Oncotype DCIS score in clinical practice.

## Introduction

Ductal carcinoma in situ (DCIS) of the breast is a non-invasive cancer encompassing nearly 25% of all breast cancer diagnoses in the mammographic era [1]. The management of DCIS traditionally consists of breast-conserving surgery (BCS) followed by whole breast radiotherapy (RT), which has been shown to reduce the risk of local recurrence (LR) [2]. Despite this, controversy exists regarding the optimal management of this condition. Many women with DCIS have a low risk of recurrence, which raises concerns of over-diagnosis and over-treatment. Conversely, a small portion of women will develop invasive recurrences (which carry a small mortality risk), raising concerns of under-diagnosis and under-treatment. Currently, several clinical and pathologic factors have been identified as prognostic markers, however, these variables alone have been unable to identify these low-risk and high-risk subgroups [3]. As a result, variability in the use or omission of RT after BCS exists, leading to unnecessary treatment of women at low-risk of recurrence and under-treatment of women who might have benefited from RT. Recently, molecular assays have been shown to provide additional prognostic information to improve risk stratification of women with DCIS, allowing for improved clinical decision-making and optimization of therapeutic ratios.

The Oncotype DX® DCIS Score is a 12-gene assay which provides an individualized 10-year estimate of the risk of LR for women with DCIS that were treated with BCS alone [4]. The DCIS score provides continuous (from zero to 100) and categorical (low, intermediate, and high-risk groups) outputs. This assay was shown to predict the risk of LR in 327 women with DCIS that took part a large prospective cohort study, Eastern Cooperative Oncology Group (ECOG) E5194 trial, assessing the risk of local recurrence in women with “low risk” clinical and pathological factors treated by BCS without RT. Women were included in the trial if they had surgical margins greater than 3 mm and were either less than 2.5 cm if Grade I/II or less than 1 cm if Grade III. The DCIS score was independently associated with the risk of any LR (HR: 2.31; p = .02) [4]. Subsequently, this score was validated in a population cohort of 571 women in Ontario with a wider range of clinical and pathologic features treated by BCS alone. Again, the Oncotype DX® DCIS score was shown to be an independent predictor of LR (HR: 1.68; p = .02) [5]. In this report, we present two cases which illustrate how the Oncotype DX® DCIS score is utilized to provide additional information beyond clinical and pathological features to both physicians and patients to optimize therapeutic decision-making.

## Case presentation

### Case 1

The first case describes a 56-year-old daycare worker with a mammographically detected left breast DCIS treated by breast-conserving surgery. The pathology report confirmed a 6 mm area of DCIS, nuclear grade 2, cribriform subtype with focal comedonecrosis. The surgical margins were clear. She had a strong family history of cardiac disease as well as a personal history of hypertension and obesity. This raised a diagnostic dilemma, as the patient would traditionally be considered a candidate for radiotherapy but was potentially at a higher risk of late radiotherapy-induced cardiac toxicity due to her underlying comorbidities. The information provided by her clinical and pathological factors suggested that she was low-risk but did not provide sufficient evidence for the omission of radiotherapy. This situation raised the concern of over-treatment with a potential for unnecessary late side effects. As such, she was offered Oncotype DX® DCIS score testing.

Three weeks after the initial assessment, the results were obtained which revealed a risk score of zero with a projected 10-year risk of LR of 9% and a 10-year risk of invasive LR of 3%. These results provided her treating oncologist with the additional information required to recommend the omission of therapy with a potential decrease in long-term radiation-induced toxicity.  

### Case 2

The second case describes a 40-year-old hospital worker with an incidental diagnosis of DCIS found after a reduction mammoplasty procedure. A pathology review confirmed a 4 mm area of DCIS, nuclear grade 2, without any necrosis. The surgical margins were unknown. Her young age and margin uncertainty put her at a higher risk of local recurrence, but the size, grade, and incidental nature of her disease were considered low-risk factors. The patient was very hesitant to have radiotherapy and was not willing to consider the alternative of undergoing a completion mastectomy procedure. Both she and her treating oncologist required additional information to help them balance the risks and benefits of adjuvant radiotherapy and to determine if a more aggressive treatment course should be considered. As such, she underwent testing with the Oncotype DX® DCIS score.

The results arrived three weeks later and indicated a risk score of 45 with a predicted 10-year risk of local recurrence of 19% and a 10-year risk of invasive local recurrence of 9%. Based on this information, the patient was able to make an informed decision to move forward with adjuvant radiotherapy.

## Discussion

Ductal carcinoma in situ is a distinct clinical entity which carries a unique diagnostic dilemma. Autopsy studies indicate that up to 15% of women that died of non-breast cancer causes were found to have occult DCIS lesions in the breast [6]. This finding raises the concern that not all DCIS lesions will be clinically relevant in a person’s lifetime. Thus, there is a risk of over-diagnosis and over-treatment, which can lead to an unnecessary risk of long-term treatment-induced side effects. Conversely, a small proportion of women with DCIS will develop a subsequent invasive breast cancer, which is associated with an increased risk of breast cancer death [7]. This subset of women is at risk for under-treatment, leading to an increased risk of recurrence, morbidity, and potential mortality. These common scenarios raise the importance of improving upon traditional methods of clinical decision-making with genomic assays.  

The above-mentioned ECOG E5194 trial found the women with “low risk” clinical and pathologic features had a 12-year rate of LR of 14.4% after treatment by BCS without RT. Many women and clinicians consider this too high and as a result, most women are recommended for and receive RT. An additional single institution prospective cohort study of 158 women with low-grade DCIS measuring less than 2.5 cm with clear surgical resection margins greater than 1 cm treated by BCS reported a 10-year rate of local recurrence of 15.6% [8]. This resulted in early termination of the study as the recurrence rate exceeded predetermined recurrence cutoffs. The Radiation Therapy Oncology Group (RTOG) 9804 trial randomized women with ‘low-risk DCIS’ to breast-conserving surgery with or without adjuvant radiotherapy. Women were eligible for the trial if they had mammographically detected DCIS that were low or intermediate grade measuring less than 2.5 cm with margins larger than 3 mm. At a median follow-up of 7.17 years, the rate of local failure was 0.9% in women that received RT compared to 6.7% in those that were treated with BCS alone [9]. These studies demonstrate the limitations of using traditional clinical and pathological features to identify a subset of patients with DCIS that are at sufficiently low risk of LR after local excision alone and highlight the requirement for additional information by genomic assays to optimize clinical decision-making.

Past studies have not been able to detect a population in which the risk is not further reduced by radiotherapy. The question then follows in regard to LR risk: how low is low enough? This answer often entails balancing the risks of recurrence (which requires additional surgery and possible mastectomy), the risks of treatment-related toxicity, and the benefits of therapy in each individual patient. Determining this therapeutic ratio may be optimized with the use of additional information. The cases highlighted in this report illustrate the value of genomic information above and beyond traditional clinicopathologic factors, providing individualized estimates of recurrence risk and allowing for optimization of patient decision-making. The first case illustrated a “low-risk” patient for whom the omission of radiotherapy (with a potential decrease in long-term toxicity) could be recommended. The Oncotype DX® DCIS score provided the patient and treating oncologist with additional information, allowing them to personalize their treatment recommendation to the patient. The second case demonstrates a “high-risk” case, which was confirmed with the genetic score, enabling the decision to administer additional treatment to lower the risk. These two cases show the utility of the Oncotype DX® DCIS score in improving risk stratification and clinical decision-making in patients with DCIS.

## Conclusions

The Oncotype DX® DCIS score is a validated, novel gene assay which provides clinicians and patients with a personalized score to estimate an individual’s risk of recurrence. This assay may improve clinical decision-making by allowing clinicians and individuals to better estimate the risks of recurrence and balance this with the potential impact of treatment, leading to enhanced therapeutic ratios for women diagnosed with DCIS. Future studies should focus on the integration of traditional clinical and pathological factors with the Oncotype DX® DCIS score to provide comprehensive, personalized risk estimates for patients.
